# The Role of Physical Activity on Mood State and Functional Skills of Elderly Women

**DOI:** 10.2174/1745017901713010125

**Published:** 2017-09-14

**Authors:** Renato Sobral Monteiro-Junior, Vinicius Dias Rodrigues, Carlos Campos, Flávia Paes, Eric Murillo-Rodriguez, Geraldo A. Maranhão-Neto, Sergio Machado

**Affiliations:** 1 Physical Education and Sport Department, State University of Montes Claros, Minas Gerais, Brazil; 2 Post Graduation Program of Health Sciences, State University of Montes Claros, Minas Gerais, Brazil; 3 Laboratory of Panic and Respiration (LABPR), Institute of Psychiatry of Federal University of Rio de Janeiro (IPUB/UFRJ), RJ - Brazil; 4 Laboratorio de Neurociencias Moleculares e Integrativas, Escuela de Medicina, División Ciencias de la Salud, Universidad Anáhuac Mayab, Yucatán, Mexico; 5 Physical Activity Sciences Postgraduate Program - Salgado de Oliveira University, Niterói, Brazil; 6 Laboratory of Physical Activity Neuroscience (LABNAF), Physical Activity Sciences Postgraduate Program (PGCAF), Salgado de Oliveira University (UNIVERSO), Niterói, RJ - Brazil; 7 Intercontinental Neuroscience Research Group, Brazil

**Keywords:** Ageing, Physical activity, Mood, Functional capacity

## Abstract

**Introduction:**

Ageing is associated with several physical, psychological and behavioral changes. These changes are closely related with global health and functional capacity in the elderly. Mood disturbances are common among the elderly and may significantly increase apathy, resulting in decreased habitual physical activity levels.

**Materials and Methods:**

The purpose of this cross-sectional study was to evaluate the mood state and functional motor capacities of elderly women engaged in a public physical activity program in Brazil and compare them with physically inactive elderly. Thirty elderly women were included in the study and categorized into two groups: physically active group, composed of participants enrolled on a public physical activity program (n = 16, 69±5 years) and physically inactive group (n = 14, 68±4 years). Total mood disturbance was assessed using the Profile of Mood States, whereas functional motor capacity was evaluated with the Sitting and Rising test. Independent t test and Mann-Whitney U] were used to compare groups.

**Results:**

The physically active group had lower total mood disturbance (p=0.02), confusion (p<0.01), tension (p<0.01), hostility (p=0.05) and fatigue (p=0.01) compared to the physically inactive group. There were no group differences regarding vigor, depression and sitting and rising performance (p>0.05).

**Conclusion:**

Lack of difference in functional motor capacity between the physically active and inactive elderly may be explained by the absence of exercise systematization in these programs.

## 1 INTRODUCTION

Ageing is widely associated with several physical, psychological and behavioral changes [1]. Furthermore, these changes are closely related with global health and functional capacity in the elderly [1-3].

Physical abilities impairment directly affects the functional motor capacity, since a person needs muscle strength and power, flexibility, balance, coordination and aerobic/anaerobic endurance in order to perform activities of daily living (ADLs) [1, 4-6]. Thus, as the aging process changes the functioning of body systems (cardiorespiratory, musculoskeletal, nervous, immune, endocrine, among others), elderly persons experience impaired functionality and disability [1, 7]. On the other hand, psychological and behavioral aspects can hinder the elderly's ability to perform ADL due to indirect mechanisms (*e.g.* low self-esteem, apathy and hypokinestetic behavior) [44]. Mood disorders such as depression are common in the elderly, resulting in reduction of the level of habitual physical activity [3, 44]. Low physical activity levels lead to diminished physical fitness and, consequently, impaired functional motor capacity. For this and other reasons, the elderly suffering from mood disorders tend to avoid exposure to instrumental ADLs, which require greater motor and cognitive complexity [8, 9]. Therefore, the factors that play a role in functional disability and mood disturbances in the elderly deserve further investigation, since physical inactivity and mental disorders are some of the factors that are associated with increased risk of mortality [10].

One of the strategies for improvement of the general aspects of health and especially the increase in physical fitness and neuroprotection is the increased level of physical activity [11], that may occur by the amount of motor activities on a daily basis, as well as by engaging in programs of regular physical activity (*i.e.*, physical exercise). From the results presented in the literature on the benefits of a healthier lifestyle, public physical activity programs have been created in Brazil to increase the level of physical activity of population and therefore prevent diseases and promote health [12, 13].

Many questions have arisen about the actual effectiveness of Brazilian public they do not follow all the recommendations for physical activity interventions and professionals and managers who run these programs lack the required technical and scientific knowledge [14, 15]. Due to the lack of systematic physical activity parameters such as intensity, it is possible that the proposed program is not enough to produce physiological and functional improvements, especially considering that many studies on this topic carried subjective measures of physical fitness through perception questionnaires or survey data [14, 16]. For this reason, it is necessary to assess both mental and physical aspects of elderly participants of physical activity programs by the Brazilian government and compare them with physically inactive elderly, in order to identify whether or not these programs are effective. Therefore, the aim of this study was to evaluate the mood state and functional motor capacities of elderly women engaged in physical activities of a public physical activity program in Rio de Janeiro and compare them with physically inactive elderly.

## 2 METHOD

### 2.1 Study Design and Ethical Aspects

This study is an analytical cross-sectional study approved by and was approved by the Research Ethics Committee of the University Salgado de Oliveira (approval protocol nº 1.220.341). All procedures performed in this study were in accordance with the ethical standards of Brazilian Council of Health and with the 1964 Helsinki declaration and its later amendments or comparable ethical standards. Participants signed a consent term and the study was approved by Ethics Committee in Research of the Universidade Salgado de Oliveira (nº 1.220.341).

### 2.2 Sample

Fifty five elderly women were recruited and evaluated at the Clinical Fisioprime Physiotherapy, located in Campo Grande, Rio de Janeiro, Brazil. Prior to the evaluation all volunteers were informed regarding the study’s procedures and signed an informed consent form. Participants were included if they had 60 years or more and excluded if they were participating in any kind of physical exercise program, use of psychotropic drugs, had any neurodegenerative disease or acute musculoskeletal injury. Twenty- five elderly women were excluded (did not met inclusion criteria or declined to participate). Thereby, a total of thirty participants were included in the study and were categorized into two groups based on physical activity levels: physically active elderly women group or physically inactive elderly women group. Participants’ physical activity level was classified according to information provided in a personal questionnaire. Participants in the physically active group (PAG) participated in a public physical activity program where they performed group-based walking activities, stretching and dance from two to three times per week engaged at least three months (n = 16). Participants from the physically inactive group (PIG) did not enroll in the public physical activity program and did not perform any kind of physical activity, except for ADLs (n = 14).

### 2.3 Assessments

#### 2.3.1 Anamnesis and Anthropometry

Before anthropometric assessment, the participants completed an interview with questions about family history, pre-existing diseases, lifestyle habits (diet, physical activity and/or exercise) and medication use. Physical activity was considered as any activity of daily living (ADL) or other activities with uncontrolled volume and intensity. If intensity and volume were controlled during any activity, it was considered physical exercise. We just collected the aforementioned information to know our sample. Moreover, we would want to certify any use of diet supplements which could influence mood. Then, they were underwent height (measured with a stadiometer) and body mass (measured with anthropometric balance) measurement.

#### 2.3.2 Total Mood Disturbance

Total Mood Disturbance (TMD) was evaluated using the *Profile of Mood States* (POMS) which assesses the mood state of each individual. Participants were asked several questions regarding how they felt in the last seven days. Each question could be scored from zero to four points. Total score represents the participant’s mood and can range from 0 to 196 points (highest score, equals worst mood). The scale comprises of encompasses six domains (tension, depression, fatigue, confusion, hostility and vigor). For this evaluation, we used the validated Portuguese version of the questionnaire with 36 questions regarding mood state [17]. Based on the score of each domain, the final score was calculated using in the equation below:

((T + D + H + F + C) - V) +100

Where,

T = tension; D = depression; H = hostility; F = fatigue; C = confusion; V = vigor.

There are no cutoff points in any domain of POMS. The scale was validated using an ordinal measurement with no classification of its score. Thus, as higher is the score, higher is the mood disorder. Internal consistency of this scale is considered high (Cronbach Alpha > 0.7).

#### 2.3.3 Functional Motor Capacity

The Sitting and Rising test (SRT) was used to assess functional motor capacity. This test evaluates the participant’s ability to sit and get up from the ground, a functional task which requires several physical aptitudes, namely muscle strength, balance and flexibility [18]. Moreover, SRT is not only associated with functional capacity but it is also a predictor of all-cause mortality [19]. Each participant initiated the test with five points. Half a point was subtracted for each loss of balance and one point for each time the participant used any body part for support. Each action (sitting down and rising up) was scored independently. Detailed scoring is shown in Table **1**.

### 2.4 Data Analysis

Descriptive and inferential statistics were used. Conceptual assumptions for conducting parametric analyzes were verified. For this we used the Shapiro-Wilk test (to check for normal distribution) and the Levene test (to check for homogeneity of variances). Despite the ordinal nature of the POMS questions, the data can be converted to a continuous variable. Therefore, if normality and homogeneity of variances were assured, parametric tests were used. Independent t Tests were used for between-group comparisons regarding TMD total and domain specific scores. Due to non-normal distribution, the Mann-Whitey U test was used for between-group comparisons regarding SRT scores. Demographic data were compared between groups to test homogeneity. All tests were performed using SPSS 17 and the level of significance was set at p≤0.05. In addition, between-group effect sizes (ES) were calculated, according to Cohen [20, 21].

## 3 RESULTS

There were no group differences regarding age (t = 0.35, df = 28, p = 0.72), body mass (t = 0.59, df = 28, p = 0.55) and height (t = 0.84, df = 28, p = 0.4). Hypertension was the most informed disease by participants. Results are shown in Table **2**. Flowchart of recruitment process is shown in Fig. (**1**).

**Fig. (2):**
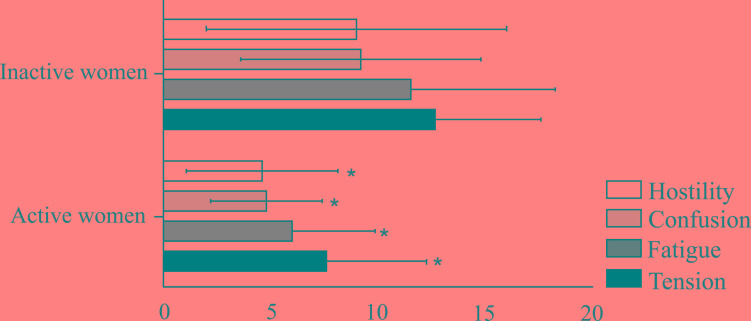
Statistically significant between groups differences in POMS subdomains. *(p≤0.05).

There were statistically significant between group differences in TMD (t = -2.41, df = 28, p = 0.02), tension (t = -2.909, df = 28, p <0.01), fatigue (t = -2.611, df = 28, p = 0.01), confusion (t = -2.800, df = 28, p <0.01) and hostility (t = -2.078, df = 28, p = 0.05), with lower scores in the physically active group in comparison to the physically inactive group for all variables. However, there were no significant differences in depression (t = -1.556, df = 28, p = 0.13), vigor (t = -0.522, df = 28, p = 0.6) and SRT sitting (U = 86.00; p = 0.25) and rising performance (U = 69.00; p = 0.06). These results are detailed in (Table **2** and Fig. **2**).

The ES ranged from trivial to large, but only the “vigor” domain was classified as trivial, while the “depression” domain was classified as moderate; and other domains and TMD were classified as large (Table **3**).

## 4 DISCUSSION

The aim of this study was to assess the mood state and functional motor capacity of elderly women engaged in a public physical activity program in Rio de Janeiro and compares them with physically inactive elderly. Our results showed that physically active elderly women had lower TMD, fatigue, confusion and hostility than physically inactive elderly. However, functional motor capacity assessed by the participants’ ability to sit down on the floor and rise up again was not statistically different between groups. There were moderate to large between group effects favoring the physically active group in all assessed outcomes, with the exception of vigor (trivial effect) and SRT Sitting subtest (small effect).

Our results regarding mood disturbance are consistent with the literature on physical activity and mental health [11, 22]. Individuals more physically active and consequently with greater physical fitness, have a lower risk of developing mood disorders, Parkinson’s and Alzheimer’s disease [11, 23-25]. Furthermore, Backmand *et al.* [26] showed that individuals with high levels of physical activity during youth had less chances of developing mood disorders late in life. Our results also corroborate other previous findings stating that high levels of habitual physical activity (frequent performed physical activity) are associated with positive affect [27]. Sarid *et al.* [28] actually found a positive association between self-reported physical activity and greater joy and vigor in the elderly. Therefore, a more active lifestyle can reduce the risk of developing health problems, mainly mood related disorders. Our results are also likely explained by the combined effects of physical activity and social interaction [29], since the active elderly women engaged in group-based physical activities. Social interaction may have been associated with motivational enhancement provided by the “neurobiological reward system”, which is directly involved in mood related processes [30].

Regarding the motor functional ability to sit and rise from floor, our findings showed no statistically difference between PAG and PIG. This finding is supported in part with other studies that found no association of habitual physical activity level with physical performance, such as muscle strength and balance, despite the relationship with other capabilities (time climbing stairs and walking speed) [31, 32]. In a study conducted for 10 years, Daly et al [33] showed that the level of habitual physical activity reduced the loss of bone density in the elderly, but did not alter muscle strength and gait speed. Therefore, the level of physical activity can be a determining factor for some functional abilities, but not all. According to the context, it is important to note that the findings of functional motor skill are in part in accordance with the few similar studies, which investigated the functional fitness of elderly participants of physical activity programs promoted by government agencies in Brazil. These studies have shown conflicting results of functional capacity of the elderly participants in these programs. For instance, Guimarães *et al*. [34] and Santos *et al.* [35] found that older people engaged in a public physical activity program only displayed significant improvement on functional motor capacity after the intervention. However, when the authors compared the differences pre-/post-program for active seniors with inactive elderly (not engaged in physical activity, but assessed at the same times of active elderly) all variables showed increased values of functional capacity.

Therefore, the disparity in the findings on the functional performance of the elderly in physical activity programs managed by public agencies in Brazil shows a necessity to revise the procedures used in conducting such activities. Although unsystematic physical activity has benefits, it is necessary to include the controlled physical exercise in such programs, so that the intensity of activity be prescribed and monitored, providing physiological adaptations and thus improving functional performance. Unsystematic physical activities, especially without intensity control, may provide equivocal results on functional capacity of the elderly [36-38]. On the other hand, a simple walking exercise program, but with intensity monitored at moderate level (50-70% of heart rate reserve) was effective in motor functional aspects [36]. In summary, in addition to physical activity (*e.g.* ADL, and active leisure activities), physical exercise (planned and repetitive activities with controlled volume and intensity) is necessary to extend the benefits and avoid functional dependence [1].

There is some evidence highlighting the functional and mental health benefits of physical activity in the elderly. However, in fact, these studies have investigated the effects of physical exercise (regular and systematic physical activity) not physical activity alone (*e.g.* ADLs) [39], while articles that differ on the functional effects actually investigated unsystematic physical activity [36, 40, 41]. Harris et al [42] showed a negative association of physically active individuals with the incidence of depression. However, the authors state that there was no control of the duration and intensity of these activities, making it difficult to understand whether the activities were systematized or not. Therefore, although the literature shows the beneficial effects of higher levels of physical activity on mental health, physical performance and more expressive neurobiological responses are more consistent when the exercise is performed [11, 22, 43]. However, according to the official Position Statement of the American College of Sports Medicine, any amount of physical activity is better than inactivity [1].

The present study has some limitations such as small sample size but mainly the study design (cross sectional). Although there was an association between physical activity and mood disturbance we cannot establish a cause-effect relationship. Therefore, our results should not be generalized.

### CONCLUSION

In conclusion, the overall mood disorder, as well as the tension, confusion, fatigue and hostility, showed a lower score in active elderly women than in physically inactive older ones. The lack of difference in functional motor capacity between the physical active and inactive elderly may be explained by the absence of physical activity systematization (exercise with controlled volume and intensity) in these programs.

## Figures and Tables

**Fig. (1):**
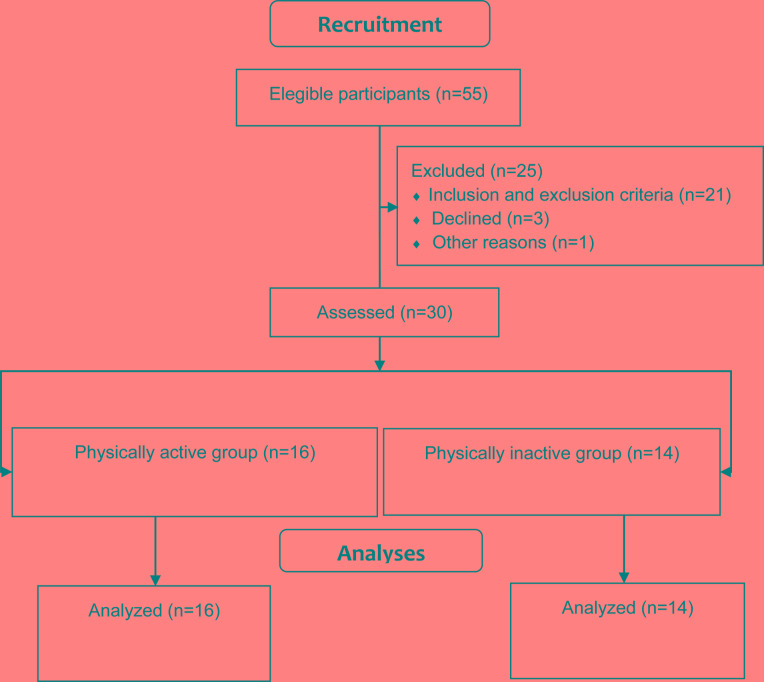
Flowchart of the recruitment process.

**Table 1:** Score of sit to raise from floor.

**Points**	**Sit**	**Raise**
5	Without Support	Without Support
4.5	1 Imbalance	1 Imbalance
4	1 Support	1 Support
3.5	1 Support and 1 Imbalance	1 Support and 1 Imbalance
3	2 Supports	2 Supports
2.5	2 Supports and 1 Imbalance	2 Supports and 1 Imbalance
2	3 Supports	3 Supports
1.5	3 Supports and 1 Imbalance	3 Supports and 1 Imbalance
1	4 Supports	4 Supports
0.5	4 Supports and 1 Imbalance	4 Supports and 1 Imbalance
0	More than 4 Supports	More than 4 Supports

**Table 2:** Comparison between groups. Mean ± standard deviation, median (minimum; maximum).

	PAG N = 16	PIG N = 14	p
Age (years)	69 ± 5	68 ± 4	0,72*
BW (kg)	69 ± 12	66 ± 11	0,55*
Height (m)	1.55 ± 0.04	1.53 ± 0.06	0,45*
TDM	121 ± 19	141 ± 24	0,02*
Tension	7.6 ± 4.7	12.7 ± 4.9	<0,01*
Fatigue	6.0 ± 3.9	11.5 ± 6.8	0,01*
Confusion	4.8 ± 2.6	9.2 ± 5.6	<0,01*
Depression	6.0 ± 4.7	9.2 ± 6.5	0,13*
Hostility	4.6 ± 3.5	9.0 ± 7.0	0,05*
Vigor	7.8 ± 6.5	9.0 ± 6.6	0,60*
TSF S	3 (0; 4)	2.7 (0; 4)	0,25^#^
TSF R	3 (0; 4)	2 (0; 4)	0,06^#^

BW: body weight; TDM: total disturbed mood; TSF: Test to sit and raise from floor; S: sit; R: raise; PAG: physically active elderly women group; PIG: physically inactive elderly women group. *Independent t Test; ^#^Mann-Whitney U Test.

**Table 3:** Effect Size between groups (mood).

	TE	Classificação
TDM	0.93	Large
Tension	1.06	Large
Fatigue	1.01	Large
Confusion	1.03	Large
Depression	0.57	Moderate
Hostility	0.81	Large
Vigor	0.18	Trivial
TSF S	0.48	Small
TSF R	0.71	Moderate

TDM: total disturbance of mood; TSF: test of sit to rise from floor; S: sit; R: rise.
